# Giant fibrovascular polyp of the esophagus - imaging techniques can localize, preoperatively, the origin of the stalk and designate the way of surgical approach: a case report

**DOI:** 10.4076/1757-1626-2-6854

**Published:** 2009-06-26

**Authors:** Panagiotis Yannopoulos, Konstantinos Manes

**Affiliations:** Esophageal Surgery Unit, “Athens Medical Center” Hospital5-7 Distomou Str, 15 125, Marousi, AthensGreece

## Abstract

Fibrovascular polyps of the esophagus are rare benign lesions that arise from the cervical esophagus and can reach very big size before they become symptomatic. Surgical excision is the treatment of choice, since endoscopic removal is not always feasible.

We present this case in order to emphasize the significance of localizing, preoperatively, the exact origin of the pedicle in planning the way of surgical approach. We consider the accurate pre-operative assessment of the origin of the pedicle essential for the proper surgical treatment of such a polyp. In respect to this, imaging provides important information concerning the exact location of the pedicle, the vascularity of the polyp and even tissue elements of the mass.

## Introduction

Fibrovascular polyps (FVP) are rare, benign “tumorlike” lesions of the esophagus, that usually remain asymptomatic. Symptoms are present when the polyp reaches a large size (resulting in their common appellation as “giant fibrovascular polyps”) and include progressive dysphagia (more than 50% of the patients), odynophagia, respiratory symptoms and the most distinctive regurgitation of a fleshy mass into the mouth which can lead in subsequent aspiration and even life-threatening asphyxia secondary to mechanical obstruction of the larynx [1,2].

Treatment consists of either endoscopic or surgical excision. If the stalk can be adequately visualized endoscopically, endoscopic ligation can be performed.

## Case presentation

The patient, a 62 years-old Caucasian male of Greek, was referred to our center due to progressive dysphagia since 2 years, an episode of regurgitation of a fleshy mass into the mouth and occasional attacks of dyspnea. Previous consultation to an Ear Nose and Throat specialist suggested a psychiatric evaluation.

**Figure 1.**
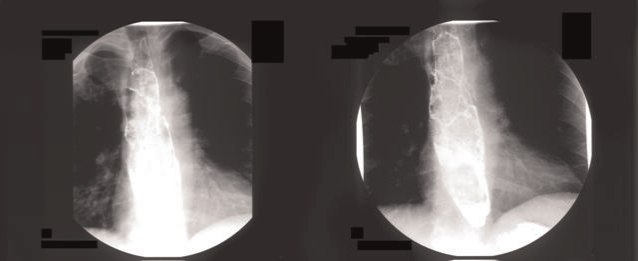
Barium esophagogram showing a filling defect of almost the entire esophagus with a sausage-like intraluminal appearance.

On admission to our hospital the patient underwent radiographic study of the esophagus using barium as contrast medium. The esophagogram demonstrated a contrast-filling defect from the cervical esophagus till the Cardioesophageal junction (Figure 1).

A mobile, elongated endoluminal polypoid mass was revealed during esophagoscopy, arising from the level of the upper esophageal sphincter and extending till just above the Cardioesophageal junction. This soft tissue polypoid mass caused a marked dilatation of the proximal and mid esophagus. Attempt to excise the polyp endoscopically was not performed due to inability to visualize adequately the base of the polyp and therefore the patient was recommended to be operated.

**Figure 2.**
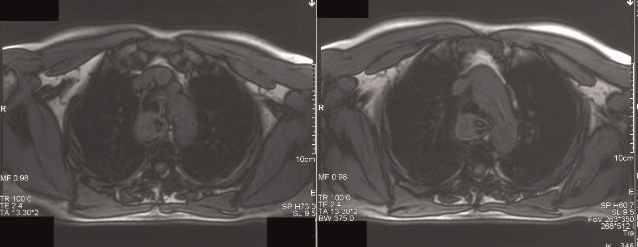
MRI T1-W axial image: a mass- like lesion is seen in the posterior mediastinum arising from the right anterior mucosal wall of the esophagus.

**Figure 3.**
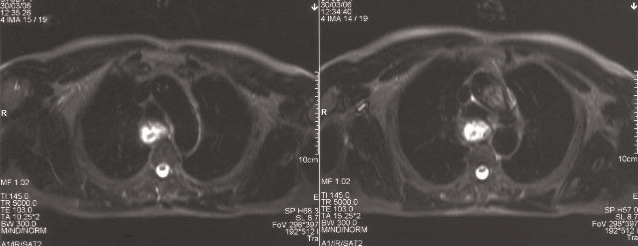
MRI T2-W axial image: the esophageal tumor appears non homogenous, with sharp margins.

MRI of the neck and thorax demonstrated that the origin of the pedicle was pointed to the right anterior mucosal wall of the cervical esophagus (Figure 2,Figure 3).

Knowing preoperatively the site of origin of the polyp, a cervical incision was decided opposite to the origin.

For this reason through a left lateral cervical approach a longitudinal esophagotomy, 5 cm in length, was performed to the left posterior esophageal wall. The mucosal origin of the stalk was completely visualized, resected and suture-closed. The mucosal defect was repaired by single interrupted absorbable stitches. The polyp was tracted and removed. The esophagotomy was sutured in a two-layered fashion.

**Figure 4.**
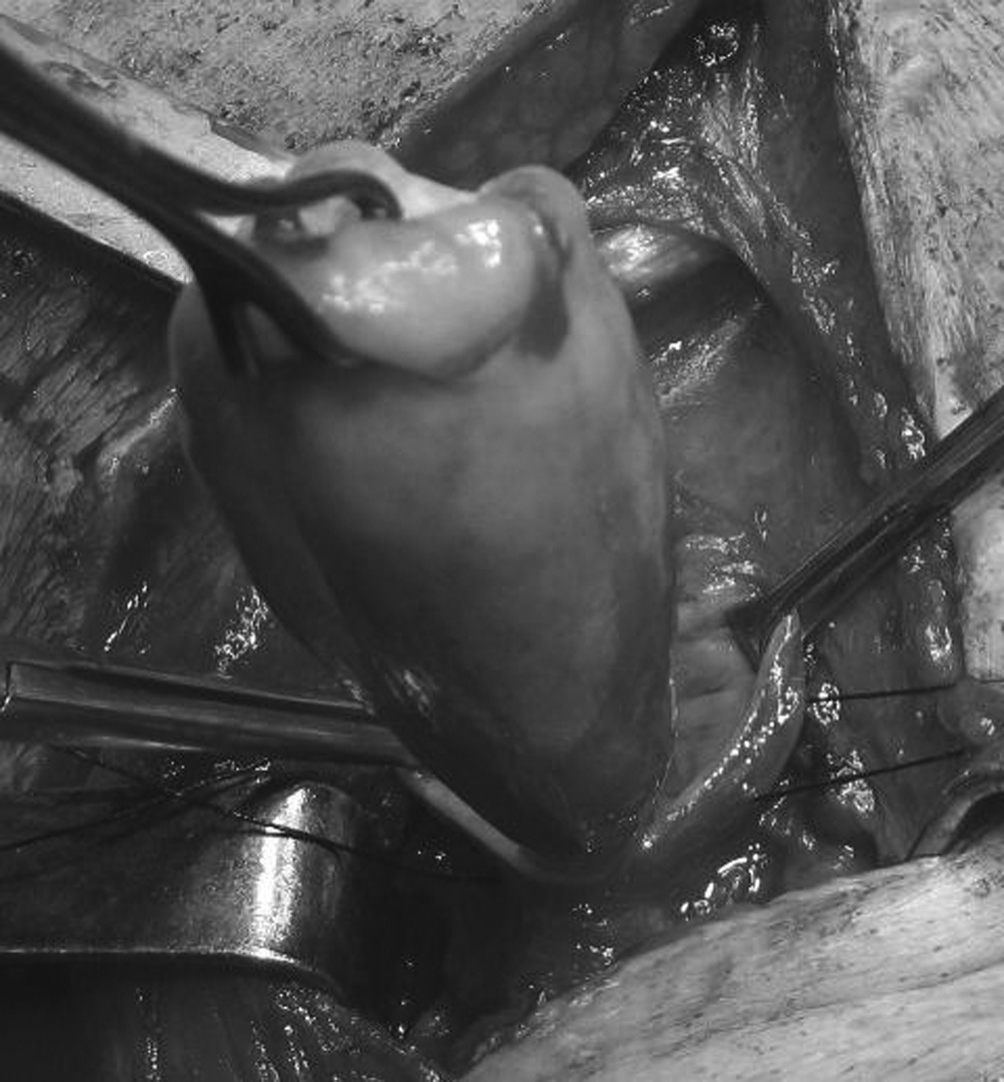
Intraoperative photograph of a large fibrovascular polyp of the esophagus. The dimension of the polyp was 10.5 × 5.5 × 3.5 cm.

The dimension of the polyp was 10.5 × 5.5 × 3.5 cm (Figure 4).

A nasogastric feeding tube was introduced and left in place for 4 days.

Histopathologic examination revealed that the specimen corresponded to a fibrovasular polyp (Figrure 4) lined with reactive squamous epithelium, with focal ischemic obstruction and necrosis of the polyp's stroma and concurrent chronic lymphonodular inflammatory reaction. There were no signs of malignancy.

The patient had an uneventful recovery period. He has been followed up for 1 year postoperatively without any sign of recurrency.

## Discussion

Although rare, fibrovascular polyps comprise the majority of benign tumor-like lesions of the esophagus characterized by the development of pedunculated intraluminal masses. Clinically, they do not present specific symptoms and are often misdiagnosed or even undiagnosed until they grow to gigantic sizes. Because these lesions are pedunculated they may have a spectacular clinical presentation, including regurgitation of a fleshy mass into the mouth. Usually these polyps arise from the cervical esophagus, inferiorly to the cricopharyngeal muscle at the Laimer's triangle, which reveals their trend to prolapse into the mouth causing the characteristic “regurgitation of a fleshy mass” [3]. The redundant mucosa in the above region of the esophagus being highly mobile is assumed to result in the polyp formation, while the propulsive act of swallowing permits its caudal extension.

Their elongated “sausage-like” characteristic appearance is believed to be the result of the traction during peristalsis and swallowing [4].

Initial diagnosis in the majority of cases is made by barium esophagogram [5]. This, usually, reveals an intraluminal contrast filling defect within a widened esophagus. The correct diagnosis can usually be suggested radiographically by the presence of a smooth, sausage-like defect with a discrete bulbous tip [6]. Many times diagnosis to be established needs additional esophagoscopy, although detection of FVP might be difficult or even impossible due to their intraluminal location and composition that looks like the esophageal mucosa [7,8]. Up to 25% of cases might be missed on endoscopy due to the normal squamous epithelium lining the polyp.

Resection, in most cases, is advocated as soon as a large fibrovascular polyp is detected to eliminate the potential risk of asphyxiation. Less usual indications for surgery include dysphagia and anemia due to gastrointestinal bleeding from the ulcerative tip of the polyp. Malignant transformation is extremely rare [9,10].

Endoscopic removal of small FVPs seems feasible. Surgical excision is mandatory whenever the polyp gets large dimensions and is performed, preferably, through a cervical esophagotomy. Surgical removal remains the treatment of choice.

Since the pedicle has to be resected under direct vision, the incision needed to expose the esophagus has to be made opposite to the site of origin of the lesion.

Making the esophagotomy to the side where the polyp originates can be disastrous, with unpleasant incidents such as severe hemorrhage and even inability to excise the polyp as a whole and leaving material that can recur.

Consequently, knowing the exact site of origin of the pedicle of the FVP is extremely important when deciding to proceed to surgical removal of such a polyp. This knowledge can be provided, preoperatively, most of the times, by modern imaging techniques (CT, MRI).

Today, planning the proper surgical approach for the resection of a giant fibrovascular polyp, has an important ally, modern imaging, which can provide important information concerning the exact location of the pedicle.

## References

[bib-001] Timmons B, Sedwitz JL, Oiler DW (1991). Benign fibrovascular polyp of the esophagus. South Med J.

[bib-002] Taff ML, Schwartz IS, Boglioli LR (1991). Sudden asphyxial death due to a prolapsed esophageal fibrolipoma. Am J Forensic Med Pathol.

[bib-003] Owens JJ, Donovan DT, Alford EL, McKechnie JC, Franklin DJ, Stewart MG, Schwartz MR (1994). Life-threatening presentations of fibrovascular esophageal and hypopharyngeal polyps. Ann Otol Rhinol Laryngol.

[bib-004] Paik HC, Han JW, Jung EK, Bae KM, Lee YH (2001). Fibrovascular polyp of the esophagus in an infant. Yonsei Med J.

[bib-005] Drenth J, Wobbes T, Bonenkamp JJ, Nagengast FM (2002). Recurrent esophageal fibrovascular polyps: case history and review of the literature. Dig Dis Sci.

[bib-006] Levine MS, Buck JL, Pantongrag-Brown L, Buetow PC, Hallman JR, Sobin LH (1996). Fibrovascular polyps of the esophagus: clinical, radiographic, and pathologic findings in 16 patients. AJR.

[bib-007] Choong CF, Meyers BF (2003). Benign esophageal tumors. Semin Thorac Cardiovasc Surg.

[bib-008] Totten RS, Stout AP, Humphreys GH, Moore R (1953). Benign tumors and cysts of the esophagus. J Thorac Sur.

[bib-009] Cokelaere K, Geboes K (2001). Squamous cell carcinoma in a giant oesophageal fibrovascular polyp. Histopathology.

[bib-010] Petry JJ, Shapshay S (1981). Squamous cell carcinoma in an esophageal polyp. Arch Otolaryngol.

